# The Psychoneuroses and Their Treatment by Psychotherapy*The Psychoneuroses and Their Treatment by Psychotherapy: By Prof. J. Dejerine, Professor of the Clinic for Nervous Diseases of the Faculty of Medicine for The University of Paris; and Dr. E. Gauckler, Ancien Interne of the Hospitals of Paris. Authorized Translation by Smith Ely Jelliffe, M. D., Ph. D., Adjunct Professor of Diseases of the Mind and Nervous System, Post Graduate School and Hospital; visiting Neurologist, City Hospital, New York. J. B. Lippincott Company, Philadelphia and London, 1913. Cloth, p. 391, price $4.00.

**Published:** 1913-09

**Authors:** 


### EDITORIAL DEPARTMENT.

DR. F. E. DANIEL, Editor DR. R. H. L. BIBB, Associate Editor

DEPARTMENTS { EUGENICS: Drs. M. Duggan and T. Y. Hull.

WOMAN'S DEPARTMENT: Mrs. F. E. Daniel.

### The Psychoneuroses and Their Treatment by Psychotherapy.*

The publishers send me this splendid book with the request that I "give it a critical review." That is rather a large order, this hot weather—90 in the shade,—and I frankly confess my inability to fill it. The subject is too deep and intricate for me to attempt it offhand, and to do it anything like justice would require days or weeks of close study, time I can not give it. Then, what's the use of trying, when the great author states so fully the meaning and intention of the work in his preface; expresses it more fully and better than I could do to save my soul. So in lieu of an attempt at a review, or a perfunctory "book notice," I shall reproduce it in full, feeling that the importance and value of the work make it "worth while," and justify the space and position here given it. Mechanically, the book is up to the Lippincott standard. I will add just a few remarks:

"When more than thirty years ago, I began to devote myself to the study of diseases of the nervous system, I was struck, from the very beginning of my practice, with the slight success which resulted from treatment of neuropaths by medicines, whether combined or not with physical measures, and little by little I was led, by personal experience, to ask myself whether it would not be wise, in the case of all patients coming under the classification of neurasthenia or hysteria, to depart from the usual therapeutic methods, and seek the cause of their disease outside of the objective symptoms which they presented.

I thus became more and more convinced that it was not the physical, but rather the moral which was the cause of all symptoms of which these patients complained, and finally, after having practiced Dr. Weir Mitchell's methods for several years, my convictions were established. In using this method of treatment, which is based practically on isolation, rest in bed, over-feeding, douches, massage, and electricity, in fact on purely physical measures, I

*The Psychoneuroses and Their Treatment by Psychotherapy: By Prof. J. Dejerine, Professor of the Clinic for Nervous Diseases of the Faculty of Medicine for The University of Paris; and Dr. E. Gauckler, Ancien Interne of the Hospitals of Paris. Authorized Translation by Smith Ely Jelliffe, M. D., Ph. D., Adjunct Professor of Diseases of the Mind and Nervous System, Post Graduate School and Hospital; visiting Neurologist, City Hospital, New York. J. B. Lippincott Company, Philadelphia and London, 1913. Cloth, p. 391, price $4.00.

was not long in discovering that unless the patient's state of mind improved the therapeutic results were far from satisfactory.

It was thus that I soon came to see that in order to treat neuropaths, with the hope of curing them, the first and most important thing was to get hold of their morale, in other words, to practice psychotherapy. This is what I have been doing for the last twenty-five years.

The influence of the morale on the physique has been known in all ages. It is in fact a popular belief that the health may be seriously affected by grief or vexation, but physicians have been, as a rule, the last persons to recognize that these might be connected with a very special class of affections, requiring particular treatment, based not on symptoms but on causes: and, without wishing to deny—at least in many cases—the accuracy of the old adage "*Mens sana in corpore sano,*" I nevertheless believe that, in the case of most neuropaths, whatever may be their symptomatology, the saying is not correct. With them, as a matter of fact, if the body is not sound it is because their morale is unhealthy, and because they have either suffered or are still suffering morally or spiritually.

As a method of general education, or moral guidance, psychotherapy is as old as the world. All philosophies, and all religions, above all, the Catholic religion—for the psychotherapist is nothing more than a confessor, or director of the lay conscience—have applied it, or are still applying it. Few, however, are the physicians who understand this, or who know how to make use of it, when they know the cause.

To be convinced of this, one only has to see what a large number of neuropaths are being subjected to some physical treatment, as if they had some true organic lesions. I am alluding to those patients whose number is legion, whom I have described under the name of false gastropaths, false enteropaths, false cardiopaths, false genitopaths, sufferers from spinal disease, and false cerebral disease; who present symptoms which often seem serious, but whose origin is wholly psychic, and who are treated every day purely and solely on the lines of symptomatic therapy, with the result that the idea becomes more firmly fixed in their minds, that there is some disease localized in the organ of which they complain. I have seen thousands of these invalids.

I hold that the physicians who understand and know how to practice psychotherapy are still very few in number. I do not, however, consider direct suggestion as a psychotherapeutic measure, either when produced more or less openly in the waking state or by means of hypnosis. Such methods have the serious defect of acting only on the subconscious, and on the cerebral automatism, and are not directed to the superior faculties of the individual.

Suggestion, though much more frequently used in hysterical cases than in troubles of neurasthenic origin, whether practiced in the waking state or during hypnotic sleep, is directed only to the symptom, and not to the cause; its action is only on the surface, it does not reach the depths. By this process one often succeeds more or less quickly in certain cases, in getting rid of a paralysis, a contracture or an anaesthesia in an hysteric. But, without taking the drawbacks into consideration, and they are very numerous, the result is very uncertain, unless, by winning the confidence and appealing to the reason of the patient, or in other words by means of psychotherapy, one succeeds in making him confess his manner of living, and explains to him how and why he fell ill, and how and why he can become cured, so that he will not relapse.

Even though these methods, which are directed only to the cerebral automatism, are sometimes successful in causing some of the objective manifestations of a hysterical condition to disappear, they are absolutely without efficiency when it comes to the very complex and intricate symptoms of a neurasthenic. For here the mental condition is wholly different. One cannot cure a false gastropath or cardiopath by a brusque command. It is a case for mental pedagogy, which often requires a long time and careful development to be effectual.

It has been stated repeatedly, and with some reason, that isolation in a sanitarium is fundamental in the treatment of the psychoneuroses. In a general way this is true, but it is not absolutely imperative. In the case of many neuropaths isolation is not necessary, and the psychotherapist need not insist on it. Isolation, in fact, is nothing but a means, without which, in many cases, it would be impossible to practice psychotherapy, and which has its special indications.

A sojourn in a sanitarium is possible only to the wealthy or those who are comfortably off, and is wholly out of the question for the poorer classes of society. But the psychoneuroses are not met exclusively among the well-to-do. Neurasthenia and hysteria are, in fact, very common among the working population of Paris, and are often found in very severe forms. I have therefore tried to introduce in the hospital the suitable conditions of treatment which one would find in a sanitarium, and for fifteen years I have established in my service at the Salpetriere an isolation and psychotherapeutic ward, where several thousand patients have been treated. The results obtained by this measure have far surpassed the hopes I had in the beginning, for they have proved quite as satisfactory, and even more rapid, than those in private practice. I will not go into the details of my methods of working in the hospital. The reader who is interested in this question will find all the necessary information in a work entitled "Isolement et Psychotherapie," published in 1904, by my pupils Camus and Paginez. I merely make, in passing, the statement that at the Salpetriere, as well as in the city, it is the moral treatment which is the cause of the success obtained.

According to some authors, particularly Dubois (of Berne), psychotherapy ought to be "rational," that is, based solely on reasoning and argument. I have always been of the opposite opinion, and I have frequently expressed myself on this subject, both in my courses at the Faculty of Medicine and in my clinical lectures at the Salpetriere. If reason and argument were sufficient to "change one's state of mind," the neuropaths would find in the writings of the moralists and philosophers, and spiritual advisers, everything they would need to reconstruct their morale, and consequently their physical well-being, and therefore they would have no need of a psychotherapist.

Reasoning by itself is indifferent. It does not become a factor of energy or creator of effort; but the moment an emotional element appears the personality of the subjects whose mentality one is seeking to modify, is moved and affected by it. According to my way of thinking, it is an error to consider both the judgment, which is a primitive phenomenon, and the impression or sentiment which follows it as psychological processes of the same nature. The impression and sentiment are nothing but the result of the more or less ready adaptation of our personality to the judgment which caused them, and though secondary are no less able to provoke reactions.

From my point of view, psychotherapy depends wholly and exclusively upon the beneficial influence of one person on another. One does not cure an hysteric or a neurasthenic nor change their mental condition by reasoning or by syllogisms. They are only cured when they come to believe in you. In short, psychotherapy can only be effective, when the person on whom you are practicing it has confessed his entire life, that is to say, when he has absolute confidence in you.

Between reasoning, and the acceptance of this reasoning by the patient, there is, I repeat, an element, on the importance of which I cannot insist too strongly; it is sentiment or feeling. It is feeling which creates the atmosphere of confidence without which, I hold, no psychotherapy is possible, that is to say,, unless reasoning produces effective action there is no *"persuasion."* I am, in fact, convinced, and have been so for a long time, that in the moral sphere the bare idea produces no effect, that is to say, the idea alone does not move one, unless it is accompanied by an emotional appeal which makes it acceptable to consciousness and thus brings about conviction. There is something analogous to faith in this, some individual element which makes the success of the phychotherapist depend upon his personality.

This is the one and only place to refer to the ancient adage—"It is faith that saves * * * or cures."

I have been aided in the collaboration of this work by one of my pupils, Dr. Gauckler, with whom I have already published several works on psychotherapy.

J. Dejerine.

Those Few Remarks.—Boiled down and divested of all scientific terms, or, as they say, "in the last analysis," this means "faith-healing," which is as old as man. Jesus is the first faithhealer of whom we have any record and if he was not a Christian Scientist, what was he? "Arise, take up thy bed and walk, thy faith has made thee whole."

Medical history is full of records of marvelous cures wrought by faith, faith in the word of the priest, religious faith being the principal element—in the psychotherapy of those days.

That wonderful cures were effected is abundantly testified; and that wonderful cures are being today wrought by the same means, faith, prayer, superstition, by the cult calling themselves Christian Scientists, can not be denied. I am personally acquainted with cases which have been so cured, that seem incredible. If they were not successful to a large degree they would not have the tremendous following—a following composed largely of the intelligent, the educated, the wealthy, who are able to build, and do build million dollar temples. And, divested of the great mass of absurdity which envelopes Mrs. Eddy's teaching, we get to the germ of truth and find that the cures are the result of psychotherapy—mental healing, effected by faith, faith in their teachers, and in Jesus' power to heal (?), and they are today practicing the psychotherapy of the days of the Roman Emperors when the church dominated the world.

Professor Dejerine, I take it, recognizes this, and would treat the thousands of cases of minor psychic disturbances which render individuals unhappy and ill, making confirmed invalids of many by a more rational (?) psychotherapy, and in his book he describes this process. He dispenses with the religious feature, of course, but uses hygiene, reason, argument, hypnotism, persuasion, suggestion and *faith in the doctor*. He would take such cases, functional neuroses, neurasthenia, the phobias—hysteria, psychoneuroses, etc.—the opprobria of medicine and the richest field of the faith-healers, out of their hands and furnish a scientific (?) therapy—just as science rescued insanity from the hands of the superstitious who regarded them as "possessed of devils" and added beatings to the curriculum. Oh, pshaw, what's the use? Read this great book.

